# Impact of Prior Infection on SARS-CoV-2 Antibody Responses in Vaccinated Long-Term Care Facility Staff

**DOI:** 10.1128/msphere.00169-22

**Published:** 2022-07-05

**Authors:** Emily N. Gallichotte, Mary Nehring, Sophia Stromberg, Michael C. Young, Ashley Snell, Josh Daniels, Kristy L. Pabilonia, Sue VandeWoude, Nicole Ehrhart, Gregory D. Ebel

**Affiliations:** a Department of Microbiology, Immunology and Pathology, Colorado State University, Fort Collins, Colorado, USA; b Columbine Health Systems Center for Healthy Aging, Colorado State University, Fort Collins, Colorado, USA; c Department of Clinical Sciences, Colorado State University, Fort Collins, Colorado, USA; University of Maryland School of Medicine

**Keywords:** COVID-19, SARS-CoV-2, correlate of protection, neutralizing antibodies, vaccines

## Abstract

Severe acute respiratory syndrome coronavirus 2 (SARS-CoV-2) emerged in 2019 and has resulted in millions of deaths worldwide. Certain populations are at higher risk for infection, especially staff and residents at long-term care facilities (LTCF), due to the congregant living setting and high proportions of residents with many comorbidities. Prior to vaccine availability, these populations represented large fractions of total coronavirus disease 2019 (COVID-19) cases and deaths in the United States. Due to the high-risk setting and outbreak potential, staff and residents were among the first groups to be vaccinated. To define the impact of prior infection on the response to vaccination, we measured antibody responses in a cohort of staff members at an LTCF, many of whom were previously infected by SARS-CoV-2. We found that neutralizing, receptor-binding domain (RBD)-binding, and nucleoprotein (NP)-binding antibody levels were significantly higher after the full vaccination course in individuals that were previously infected and that NP antibody levels could discriminate individuals with prior infection from vaccinated individuals. While an anticipated antibody titer increase was observed after a vaccine booster dose in naive individuals, a boost response was not observed in individuals with previous COVID-19 infection. We observed a strong relationship between neutralizing antibodies and RBD-binding antibodies postvaccination across all groups, whereas no relationship was observed between NP-binding and neutralizing antibodies. One individual with high levels of neutralizing and binding antibodies experienced a breakthrough infection (prior to the introduction of Omicron), demonstrating that the presence of antibodies is not always sufficient for complete protection against infection. These results highlight that a history of COVID-19 exposure significantly increases SARS-CoV-2 antibody responses following vaccination.

**IMPORTANCE** Long-term care facilities (LTCFs) have been disproportionately impacted by COVID-19, due to their communal nature, the high-risk profile of residents, and the vulnerability of residents to respiratory pathogens. In this study, we analyzed the role of prior natural immunity to SARS-CoV-2 in postvaccination antibody responses. The LTCF in our cohort experienced a large outbreak, with almost 40% of staff members becoming infected. We found that individuals that were infected prior to vaccination had higher levels of neutralizing and binding antibodies postvaccination. Importantly, the second vaccine dose significantly boosted antibody levels in those that were immunologically naive prior to vaccination, but not in those that had prior immunity. Regardless of the prevaccination immune status, the levels of binding and neutralizing antibodies were highly correlated. The presence of NP-binding antibodies could be used to identify individuals that were previously infected when prevaccination immune status was not known. Our results reveal that vaccination antibody responses differ depending on prior natural immunity.

## INTRODUCTION

Severe acute respiratory syndrome coronavirus 2 (SARS-CoV-2), the virus responsible for coronavirus disease 2019 (COVID-19), has resulted in over 400 million infections worldwide, with 78 million occurring in the United States (1). Infections in staff and residents of long-term care facilities (LTCFs) account for ~2 million of those infections and represent 16% of all COVID-19 deaths in the United States (2). LTCFs are high-risk environments due to their congregant living setting and high proportions of residents with multiple comorbidities, including diabetes and lung and heart disease (3–5). Because of this, LTCFs have been at the forefront in surveillance testing to detect infections in staff and residents before they spread and cause outbreaks (6, 7). Additionally, staff and residents at LTCFs were prioritized as one of the first groups to receive vaccines once available, and as of February 2022, over 80% of staff and residents were fully vaccinated nationally (2).

Due to the high numbers of cases in LTCFs prior to vaccines and other preventative measures, many staff and residents became infected during 2020 and 2021, with some facilities reporting infection and seroprevalence rates as high as 40% (8–11). Therefore, there were two immunologically distinct populations of individuals receiving vaccines: those that were naive, with no evidence of a prior infection (seronegative), and those with preexisting immunity, having either a documented prior infection or serological evidence of prior infection (seropositive). Early work examined the role of preexisting immunity in the levels of binding antibodies up to 4 weeks following a single dose of an mRNA vaccine (both Pfizer and Moderna) and found that the levels were higher in those that were seropositive (12). Additional work has evaluated longer-term responses after two vaccine doses and similarly found that those with prior infections generated higher levels of binding antibodies (13–15). Most of these studies did not measure polyclonal antibody neutralization of live SARS-CoV-2 virus and instead used pseudotyped virus or receptor blocking assays as surrogates of true neutralization.

Staff at a local long-term care facility (LTCF), in parallel with their weekly SARS-CoV-2 nasal surveillance quantitative reverse transcriptase PCR (qRT-PCR) testing, provided blood samples for antibody analyses (8). This facility experienced a SARS-CoV-2 outbreak in September 2020 prior to vaccine availability, resulting in infection and seroconversion of almost 35% of the staff members (8). In January 2021, a Pfizer vaccine clinic was provided at their workplace, with the second dose provided 3 weeks later in early February. Vaccines were not required at that time, though vaccination is now required with rare exceptions (16). As of 30 January 2022, 96% of staff and 97% of residents at this facility were fully vaccinated, slightly higher than Colorado statewide averages (92% of staff and 93% of residents, respectively) (2). We collected and analyzed sera from staff at this facility from August to December 2020 (8). We found that during an outbreak at the facility, many staff (~30%) became infected and subsequently seroconverted, generating neutralizing, spike-binding, and RBD-binding antibodies. Here, we report serum antibody levels detected in samples collected from February through September 2021 to examine humoral immune response duration. We characterized antibody neutralization and binding to the receptor-binding domain (RBD; contained within the spike protein component of the vaccine) and nucleocapsid (NP; not present within the mRNA vaccines). We found that individuals with a prior SARS-CoV-2 infection had higher postvaccination neutralizing and RBD- and NP-binding antibodies than those that were seronegative prior to vaccination and that individuals that were never infected with SARS-CoV-2 did not harbor anti-NP seroreactivity.

## RESULTS

### Neutralizing serum antibodies increase following vaccination regardless of prevaccination immune status.

**FIG 1:**
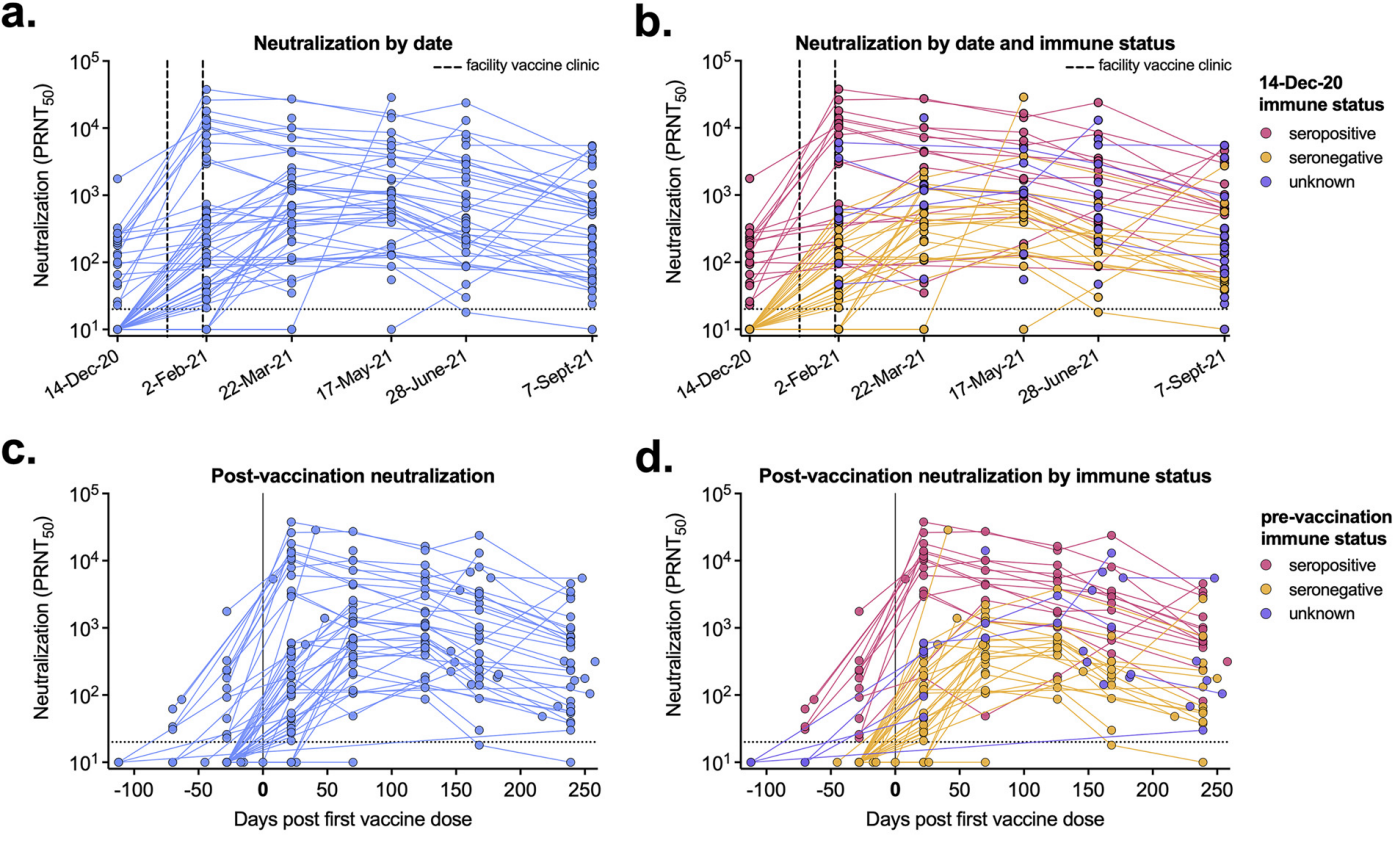
Postvaccination serum neutralizing levels vary by prior infection. (A to D) Neutralization titers (PRNT_50_) for each serum sample are shown by blood sample collection date (92 participants, 260 samples total) (A, B) or by days post-first vaccine dose (68 participants, 226 samples total) (C, D). (B, D) Serum sample data are labeled based on participants’ prevaccination immune status. (B) Pink, seropositive, 23 participants, 86 samples; orange, seronegative, 38 participants, 127 samples; purple, unknown immune status, 31 participants, 47 samples. (D) Pink, seropositive, 16 participants, 70 samples; orange, seronegative, 32 participants, 121 samples; purple, unknown immune status, 20 participants, 35 samples. Dashed lines represent the limit of detection (PRNT_50_ = 20). Samples without neutralization detected are plotted at half the limit of detection (PRNT_50_ = 10).

By February 2021, the neutralizing antibody levels of most individuals increased following one or two doses of vaccine. By mid-March 2021, almost all participating staff had detectable neutralizing antibody levels (Fig. 1a). Based on prior surveillance testing and antibody analyses (8), we stratified individuals based on their December 2020 immune status as either seropositive (immune), seronegative (naive), or unknown. On average, immune individuals had higher levels of neutralizing antibodies than those that were seronegative (Fig. 1b). Not all individuals within this cohort were vaccinated, and in those that were not, the neutralizing antibodies detected resulted from natural infection and not vaccination. We next focused on vaccinated individuals and analyzed the neutralizing antibody response based on time post-first vaccine dose (ranging from December 2020 to August 2021). When analyzed by days post-first vaccine dose, we demonstrated a rapid increase of neutralizing antibody levels (Fig. 1c). When stratified by prevaccination immune status, individuals that were previously infected had higher levels of neutralizing antibodies postvaccination than individuals that were seronegative prior to vaccination (Fig. 1d).

### RBD- and NP-binding antibody levels after vaccination.

**FIG 2:**
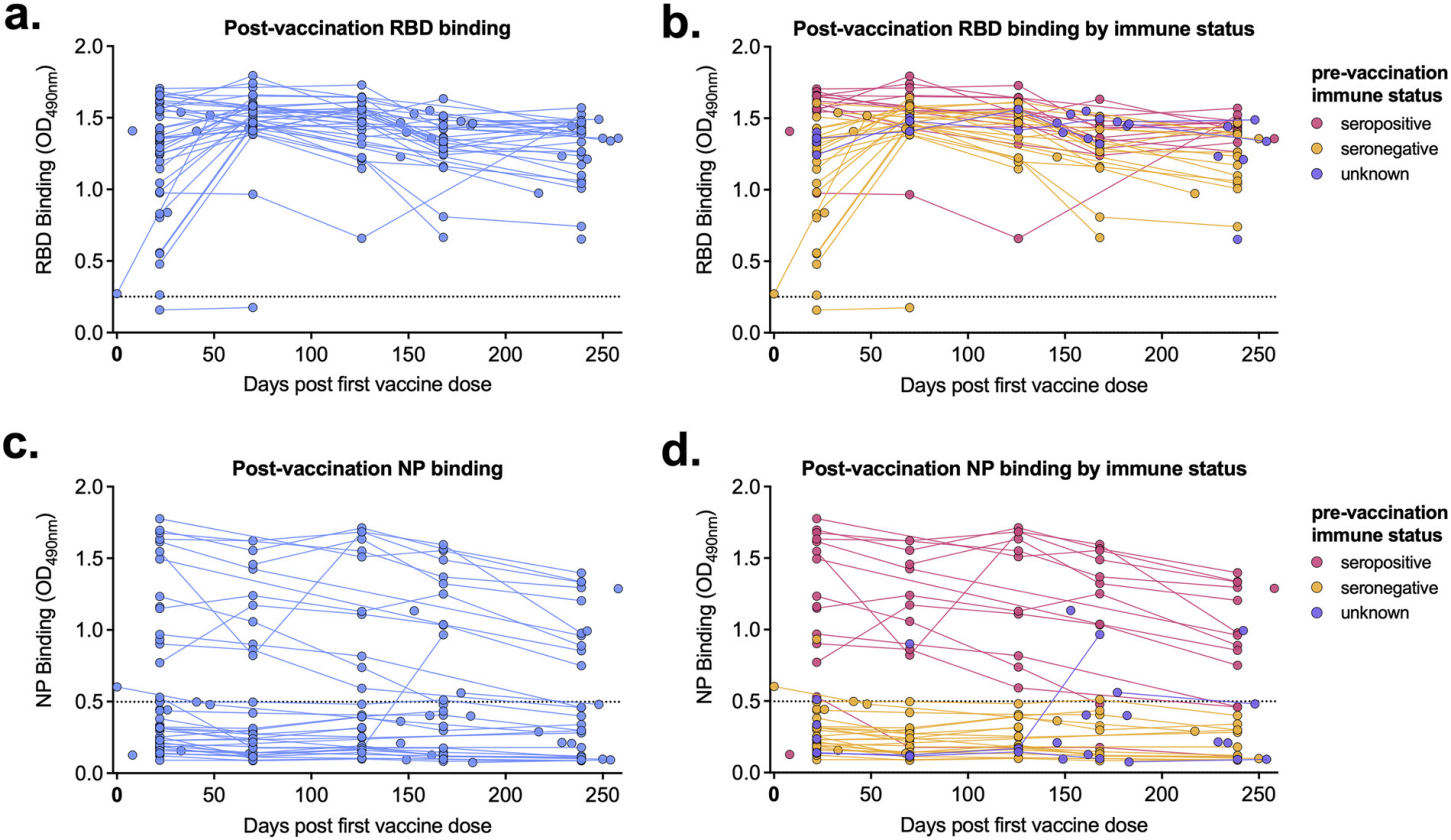
Postvaccination receptor-binding domain (RBD)- and nucleoprotein (NP)-binding levels are higher in previously infected individuals. (A to D) RBD-binding (A, B) and NP-binding (C, D) levels for each serum sample are shown by days post-first vaccine dose (68 participants, 178 samples total). (B, D) Serum sample data are labeled based on participants’ prevaccination immune status. Pink, seropositive, 17 participants, 61 samples; orange, seronegative, 31 participants, 87 samples; purple, unknown immune status, 20 participants, 30 samples. Dashed line represents background level for each assay. OD_490_, optical density at 490 nm.

We next measured receptor binding domain (RBD)- and nucleoprotein (NP)-binding antibody levels following vaccination in our cohort participants. RBD-binding antibodies reached their maximum levels in all individuals by day 70 postvaccination and gradually decreased over the next 6 months (Fig. 2a). Seropositive individuals had slightly higher RBD absorbance values than those that were immunologically naive prior to vaccination, though this enhancement was not as marked as that of neutralizing antibody levels (Fig. 2b). Participants in our cohort received either the Pfizer or Moderna mRNA vaccine, both of which encode the viral spike protein (which contains the RBD). Therefore, as expected, participants with NP-reactive antibodies (Fig. 2c) were previously infected with SARS-CoV-2 (Fig. 2d).

### Postvaccination antibody levels are higher in individuals with preexisting immunity.

**FIG 3:**
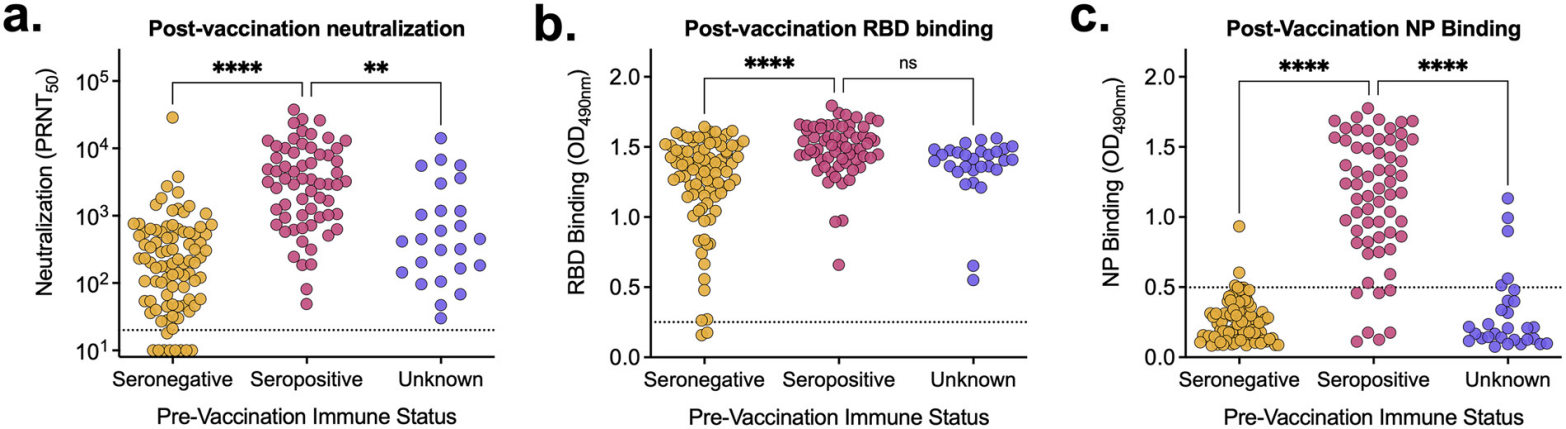
Prevaccination immune status impacts postvaccination antibody levels. (A to C) All postvaccination neutralization titers (A) and RBD-binding (B) and NP-binding (C) values were aggregated and stratified based on prevaccination immune status. Pink, seropositive, *n* = 90; orange, seronegative, *n* = 63; purple, unknown immune status, *n* = 25. (A) Dashed line represents the limit of detection. Samples without neutralization detected are plotted at half the limit of detection (PRNT_50_ = 10). (B, C) Dashed line represents the background level for each assay. Tukey’s multiple-comparison one-way analysis of variance (ANOVA) was used to determine statistical significance. **, *P* < 0.01; ****, *P* < 0.0001; ns, not significant.

When compiling all samples collected postvaccination (including those after only the first dose), we saw that seropositive prevaccination individuals had significantly higher (*P* < 0.0001) levels of neutralizing and RBD-binding antibodies than seronegative individuals (Fig. 3a and b). Since there is no nucleoprotein component in the vaccine, it is not surprising that only individuals that experienced a SARS-CoV-2 infection prior to vaccination had detectable NP antibodies (Fig. 3c). From these results, we can presume that individuals with unknown prevaccination immune status (Fig. 3c, purple) with detectable NP-binding antibodies (three participants each with a single sample) had experienced a SARS-CoV-2 infection prior to vaccination.

### Impact of second vaccine dose on antibody levels is dependent on prevaccination immune status.

**FIG 4:**
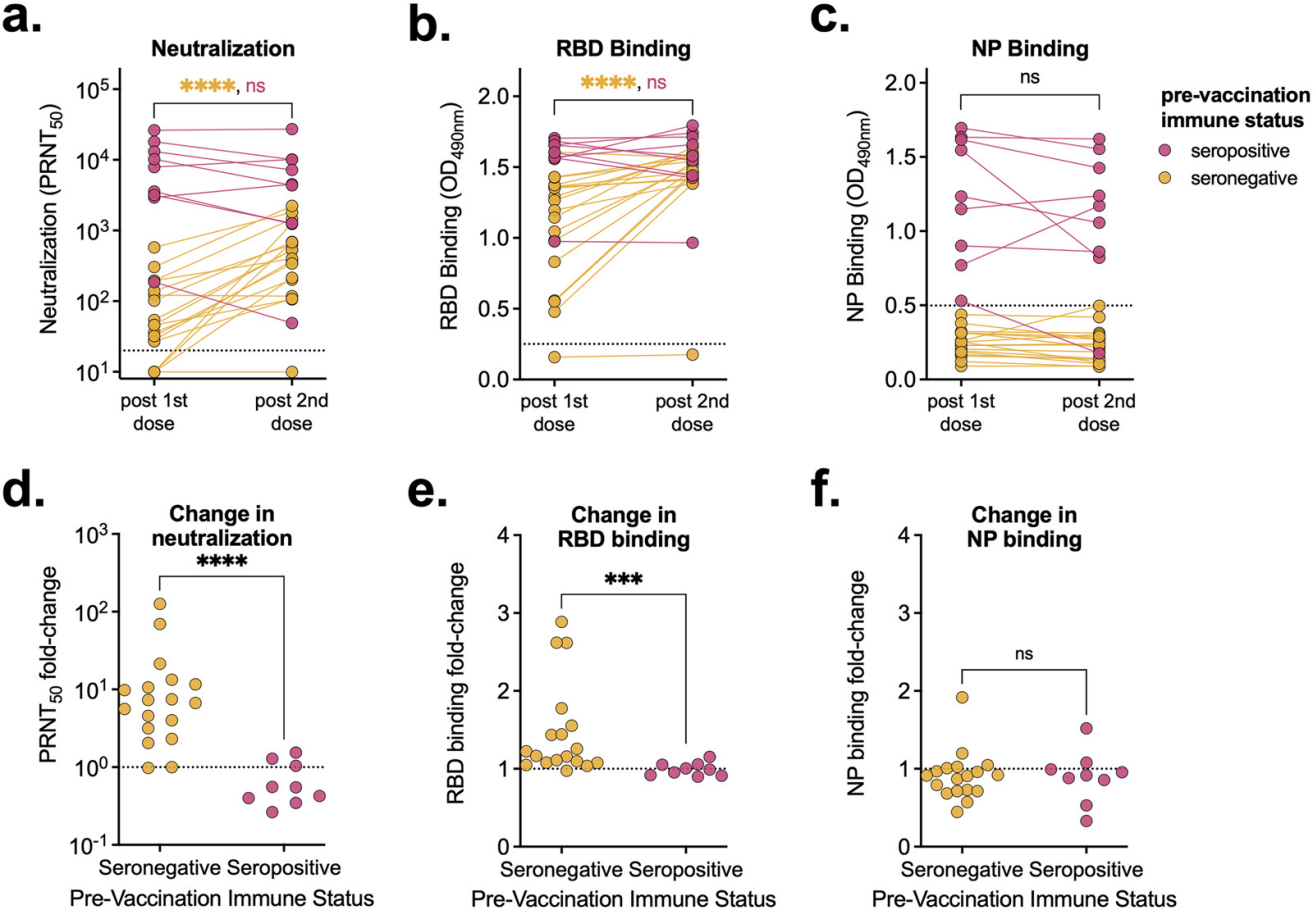
A second vaccine dose only increases antibody levels in seronegative individuals. (A to C) Neutralization titers (A) and RBD-binding (B) and NP-binding (C) values of serum samples from individuals following their first and second vaccine doses (3 weeks after the first dose and 7 weeks after the second dose), stratified by prevaccination immune status (seropositive, *n* = 9; seronegative, *n* = 18). (D to F) Fold changes between neutralization titers (D) and RBD-binding (E) and NP-binding (F) values relative to levels following participants’ first vaccine doses. (A) Dashed line represents the limits of detection. Samples without neutralization detected are plotted at half the limit of detection (PRNT_50_ = 10). (B, C) Dashed line represents the background level for each assay. Mann-Whitney test was used to determine statistical significance. ***, *P* < 0.001; ****, *P* < 0.0001; ns, not significant.

A subset of the cohort with known serostatus prior to vaccination provided blood samples following both their first and second vaccine doses. We compared the levels of neutralizing, RBD-binding, and NP-binding antibodies across these two time points and cohorts and looked at relative changes in antibody levels (Fig. 4). In individuals that were immunologically naive prior to vaccination, neutralizing and RBD-binding antibody levels increased significantly between the first and second doses (*P* < 0.001) (Fig. 4a and b). Importantly, some individuals did not have detectable neutralizing antibodies until after their second dose. In contrast, in previously infected individuals, neutralizing and RBD-binding antibody levels did not increase significantly following their second dose (Fig. 4a and b). Additionally, vaccination did not alter NP-binding antibody levels regardless of prevaccination immune status (Fig. 4c). In seronegative individuals, following the second vaccine dose, neutralizing and RBD-binding antibody levels increased significantly (average increases of 17-fold and 1.5-fold, respectively) (Fig. 4d and e). Conversely, in prevaccination seropositive individuals, on average, neutralizing, RBD-binding, and NP-binding antibody levels did not change following the second vaccine dose (0.7-, 1-, and 0.9-fold changes, respectively) (Fig. 4d, e, and f).

### Relationship between neutralizing and binding antibodies in vaccinated individuals.

**FIG 5:**
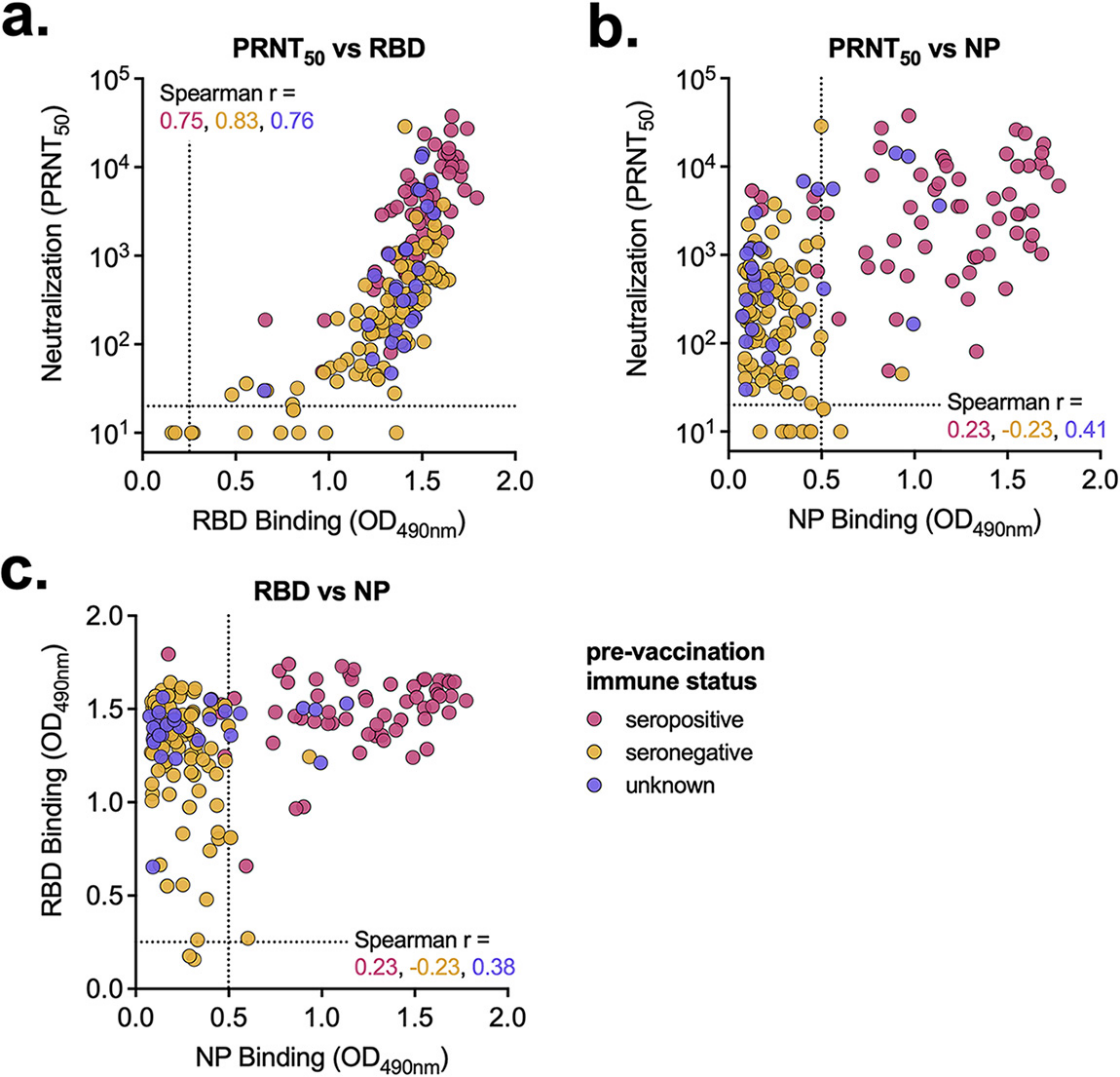
Vaccine-elicited antibody levels are similarly correlated regardless of immune status. (A to C) Postvaccination serum samples were compared by neutralization versus RBD binding (A), neutralization versus NP binding (B), and RBD binding versus NP binding (C). Serum sample data are labeled based on prevaccination immune status. Pink, seropositive, 17 participants, 61 samples; orange, seronegative, 33 participants, 91 samples; purple, unknown immune status, 18 participants, 26 samples. (A) Dashed line represents the limit of detection. Samples without neutralization detected are plotted at half the limit of detection. (B, C) Dashed line represents the background level for each assay. Spearman *r* values for each group (seropositive, seronegative, and unknown) are noted.

We next compared the relationship between neutralizing and binding (both RBD and NP) antibodies in vaccinated individuals (including samples collected after just the first dose), stratified by prevaccination immune status. We saw a strong relationship (*r* > 0.75) between neutralizing titer and RBD-binding antibody absorbance regardless of immune status (Fig. 5a). Because NP-binding antibodies are only found in individuals that experienced a natural SARS-CoV-2 infection, the relationships with NP antibodies (both 50% plaque reduction neutralization titer [PRNT_50_] versus NP, and RBD versus NP) were poorly correlated (*r* < 0.45) in postvaccination serum samples (Fig. 5b and c).

### Breakthrough infection in a vaccinated individual with high levels of antibodies.

**FIG 6:**
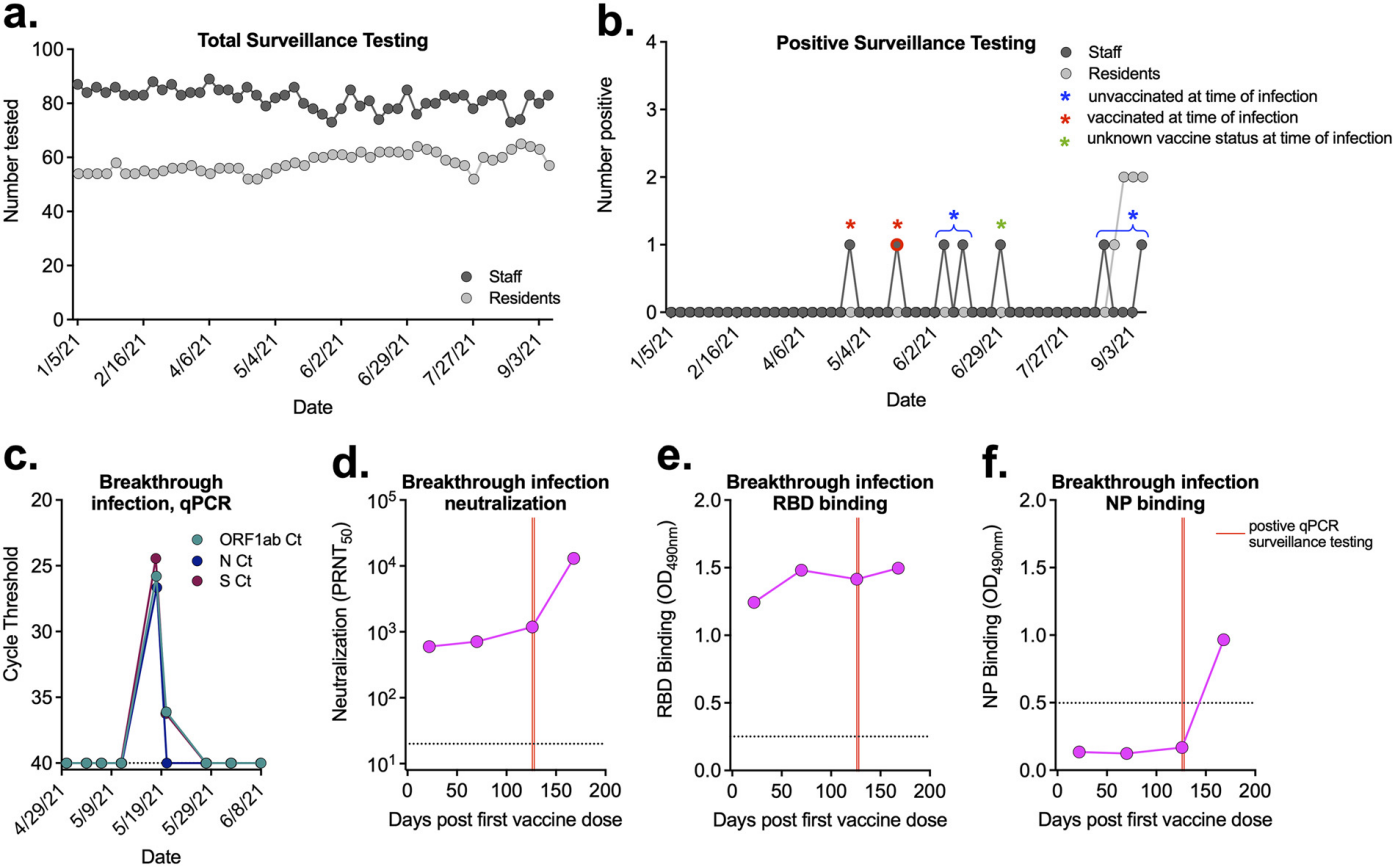
Breakthrough infection in a vaccinated individual. (A) Total surveillance testing on staff and residents at the LTCF each week. (B) Number of staff and residents that tested positive each week. Blue asterisks indicate staff members that were unvaccinated at time of infection (brackets show positive tests from the same individual). Red asterisks indicate staff members that were vaccinated prior to infection. Green asterisk represents a staff member with unknown vaccine status at time of infection. Detailed data for individual represented by symbol with red outline are given in panels C to F. (C) qRT-PCR surveillance testing for three viral targets from the individual with a breakthrough infection that was seronegative prior to vaccination. (D to F) Neutralization titers (D) and RBD-binding (E) and NP-binding (F) values postvaccination and pre- and post-breakthrough infection. Red lines correspond to date of positive qRT-PCR surveillance testing. (C, D) Dashed lines represent limits of detection. (E, F) Dashed line represents the background level for each assay.

As part of mandated Centers for Medicare & Medicaid Services (CMS) surveillance testing (16), staff and residents were tested weekly for SARS-CoV-2 viral RNA via nasal swabs (Fig. 6a). During the study time period, there were seven positive tests from five unique individuals among staff. Each of these infections occurred during a time when no residents tested positive, suggesting that the staff members were not infected at work (Fig. 6b). Two of these individuals were unvaccinated at the time of their infection, and one had unknown vaccine status (Fig. 6b). Two individuals in the cohort experienced a breakthrough infection (vaccinated at time of infection); however, only one provided sera samples for antibody analyses (Fig. 6b). This individual was seronegative prior to vaccination (no evidence of neutralizing antibodies, nor had they ever tested positive during weekly surveillance testing) and received both vaccine doses in early 2021. In May 2021, this individual experienced an asymptomatic acute breakthrough infection prior to the introduction of the Omicron variant (Fig. 6c). There was no evidence that their antibody levels had waned prior to infection (Fig. 6d and e). Their neutralizing antibody levels increased rapidly following infection (Fig. 6d), whereas their RBD-binding antibodies did not (Fig. 6e). The detection of anti-NP antibodies confirmed the breakthrough infection (Fig. 6f).

## DISCUSSION

Early following SARS-CoV-2 vaccine approval, it was unclear whether both doses of the mRNA vaccine would be necessary for individuals that had previously been infected to achieve full protection (17). It was predicted that the first dose would boost humoral immunity acquired from a natural infection. Multiple studies have demonstrated that in previously infected, seropositive individuals, a single vaccine dose is sufficient to generate robust immune responses (both humoral and cellular), often to levels higher than in naive individuals that received two vaccine doses (18–20). Our data confirm that individuals with a prior infection generate a robust neutralizing antibody response that is not further increased upon a second dose. These results have led to calls for a single-dose vaccine regimen in previously infected individuals to stretch vaccine supplies, improve worldwide vaccine access, and increase vaccine uptake among hesitant COVID-19 survivors (21–24).

Conversely, in seronegative individuals, antibody levels increased significantly following a second vaccine dose (18–20). Three individuals in our cohort did not generate neutralizing antibodies until after the second vaccine dose, and one individual never seroconverted following vaccination. It is therefore critical that individuals without prior infection receive the full vaccination course to ensure maximum immune response (25).

Neutralizing and binding antibody levels are being investigated as possible correlates of protection, as they are highly correlated with vaccine efficacy across diverse cohorts and vaccine platforms (26–28). There are reports describing breakthrough infections postvaccination, likely due to reduced/waning antibody levels and timing postvaccination (29–32). The breakthrough infection that occurred in our cohort was in an individual with high neutralizing antibody levels, similar to other recent reports (33, 34). These data suggest that while antibody levels may be broadly predictive of vaccine efficacy, they are not sufficient as a singular correlate of protection in all individuals.

Our work, along with that of others (35–37), describes the use of nucleoprotein antibody detection as a tool to identify natural infection using serum collected postvaccination. This assay could be used to further define and refine correlates of protection or to generate a better predictor of breakthrough risk by stratifying data from postvaccination serum samples according to whether the donors had or had not been previously infected. Importantly, this strategy is only effective in individuals that received a vaccine without a nucleocapsid component (Pfizer, Moderna, etc.), as opposed to inactivated-whole-virus vaccines (or other similar vaccine platforms) containing nucleocapsid, such as Sinovac.

There remain many unknowns regarding the immune response following COVID-19 infection, vaccination, booster, and breakthrough infection (38–40). Boosters, which have been widely accessible in the United States, combat waning immunity by boosting preexisting adaptive immunity (both humoral and cellular), furthering protection against severe disease (41). There is relatively high booster uptake among staff and residents of LTCFs in Colorado (76% and 40% of residents and staff with boosters, respectively), with slightly higher rates in the facility described in this paper (80% residents, 44% staff) (2). Despite high vaccination and booster rates, the Omicron variant seems to efficiently evade vaccine-elicited immunity (42, 43). These results suggest that additional boosters and variant-specific boosters might be required to maintain long-term immunity against SARS-CoV-2 (44).

## MATERIALS AND METHODS

### Human specimens.

This study was approved by the Colorado State University Institutional Review Board under protocol number 20-10057H. Participation in providing blood samples was voluntary. Participants gave consent and were enrolled and informed of test results. Staff represented a range of job classifications, including those in direct patient care roles (e.g., nurses) and nondirect patient care roles (e.g., administrative). Participation in the antibody component of the study was entirely voluntary, and approximately 55% of staff members provided serum samples at least once during the study.

### Serum sample collection.

Whole blood was collected in BD Vacutainer blood collection tubes and allowed to clot at room temperature for at least 30 min. The tubes were spun at 1,300 × *g* for 10 min to separate sera from clotted blood. Sera were aliquoted, heat inactivated at 56°C for 30 min, and stored at 4°C.

### Viruses and cells.

Vero cells (ATCC-81) were maintained in Dulbecco modified Eagle medium (DMEM) with 10% fetal bovine serum (FBS) and 1% antibiotic/antimycotic at 37°C and 5% CO_2_. SARS-CoV-2 virus (2019-nCoV/USA-WA1/2020 strain) was used to infect Vero cells for 3 days, and supernatant was harvested, centrifuged at maximum speed for 10 min to pellet cell debris, aliquoted into single-use aliquots, and stored at −80°C until use.

### Neutralization assay.

A standard plaque reduction neutralization test (PRNT) was performed as previously described (8). Briefly, diluted serum samples were mixed with virus, incubated for 1 h at 37°C, added to a Vero cell monolayer, incubated for an additional hour at 37°C, and then overlaid with tragacanth medium and incubated for 2 days. Cells were fixed and stained with ethanol and crystal violet, and plaques counted manually.

### RBD and NP ELISA.

Binding assays were performed as described previously (8). Briefly, 96-well plates were coated with SARS-CoV-2 protein (RBD and NP from Sino Biological) and blocked with nonfat dried milk, and diluted serum was added. Plates were washed, and a secondary anti-human IgG–horseradish peroxidase-conjugated secondary antibody was added. Plates were developed and read at 490 nm on a spectrophotometer.

### Surveillance qRT-PCR testing.

Mandatory surveillance testing was performed on staff and residents as previously described (8, 9). Briefly, nasal swabs were collected and processed, viral RNA extracted, and quantitative reverse transcriptase PCR (qRT-PCR) performed using the Thermo Fisher Scientific TaqPath COVID-10 combo kit, under U.S. FDA Emergency Use Authorization (45).
